# Pulmonary consolidation alters the ultrasound estimate of pleural fluid volume when considering chest drainage in patients on ECMO

**DOI:** 10.1186/s13054-022-04018-9

**Published:** 2022-05-18

**Authors:** Martin Balik, Masego Candy Mokotedi, Michal Maly, Michal Otahal, Zdenek Stach, Eva Svobodova, Marek Flaksa, Jan Rulisek, Tomas Brozek, Michal Porizka

**Affiliations:** 1 Department of Anaesthesiology and Intensive Care, 1st Faculty of Medicine, Charles University and General University Hospital in Prague, U Nemocnice 2, 12808 Prague 2, Czech Republic; 2 Department of Radiology, Institute of Clinical and Experimental Medicine, Videnska 1958/9, 14021 Prague 4, Czech Republic

## Dear Editor,

**Fig. 1:**
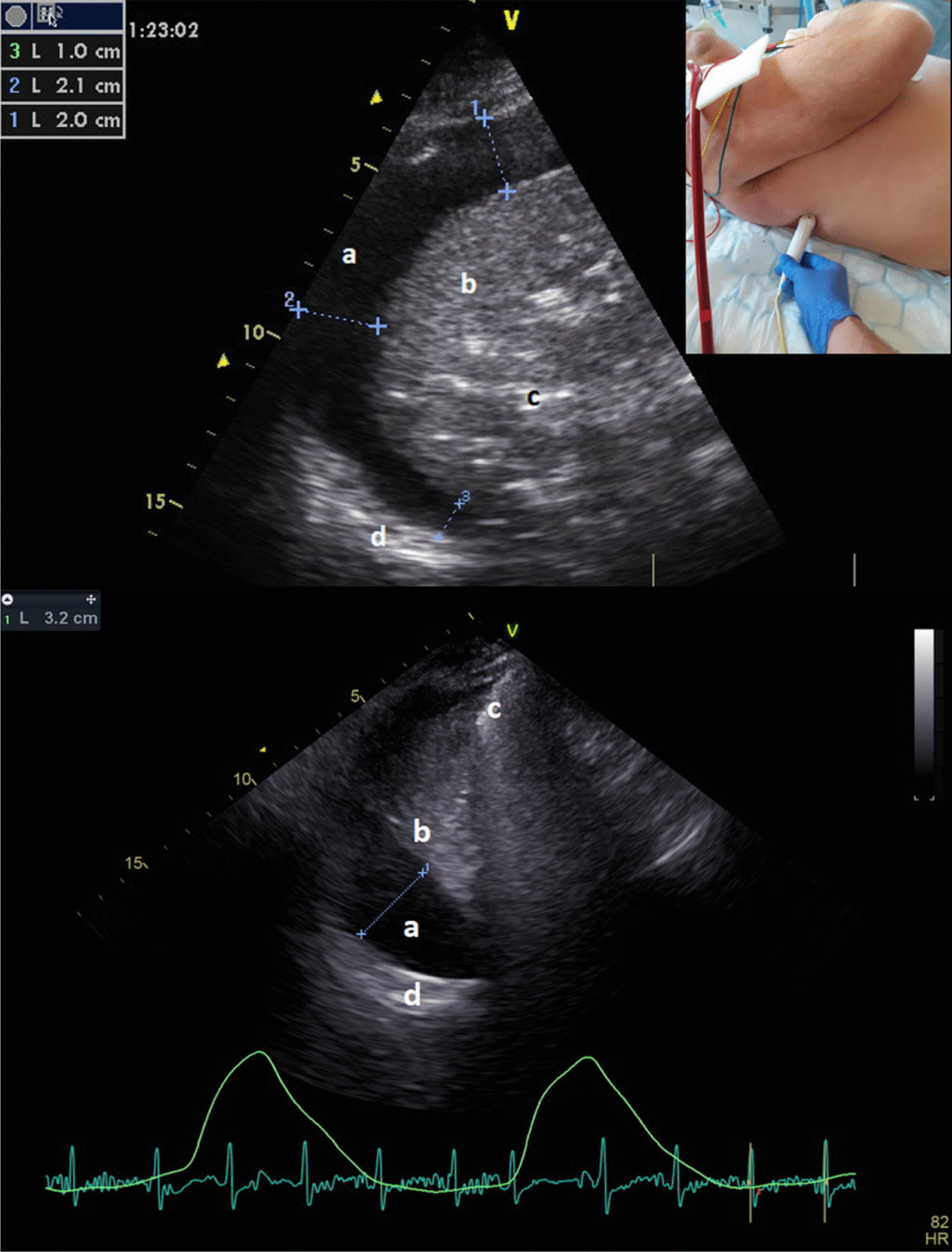
Phased array transducer scanning in the transverse plane above the right lung base in the posterior axillary line. It is positioned at the expected chest drainage spot allowing measurements of the depth of pleural layers in the intercostal space which contributes to the safety of the procedure (upper). Consolidated right lower lobe in a patient on ECMO with circumferential effusion that separates the pleural layers paravertebrally (3 = Psep) 10 mm, dorsally (2 = Dsep) 21 mm and laterally (1 = Lsep) 20 mm (a pleural fluid, b consolidated lung parenchyma, c bronchogram, d rib). The drained volume of pleural fluid was 980 ml (middle). For comparison the original method of pleural fluid estimation [1] in another non-ECMO cardiac patient (bottom). The maximum separation of 32 mm at the lung base is multiplied by 20 giving a pleural volume estimate of 640 ml. Note no pleural separation and aerated lung under the posterolateral chest wall (a pleural fluid, b compressed lung parenchyma, c aerated lung, d rib)

The estimation of pleural fluid volume evaluated by bedside chest ultrasound (CUS) helps to decide on pleural drainage. The method of multiplying the maximum transverse pleural separation at the base of the lung in millimetres by 20 has been formulated and independently verified in mechanically ventilated patients [1, 2]. The method presumes an aerated lung floating in pleural fluid with expected compression of the basal segments and largest pleural separation at the lung base (Fig. 1). The calculation may fail in extensively consolidated and less buoyant lungs encircled by pleural fluid which may be found in severe ARDS treated, in addition to IPPV, with the extracorporeal membrane oxygenation (ECMO). The degree of lung consolidation evaluated as alveolo-interstitial syndrome can also be diagnosed and quantified by a bedside ultrasound [3]. We tested the hypothesis that in patients with extensive lung consolidation with scores 3 (coalescent B lines) to 4 (consolidation) in all lung regions, the established method of estimating pleural fluid volume may produce a large prediction error. Multiple pleural separation measurements may be more useful to provide an accurate quantification of pleural fluid.

Pleural drainage data were prospectively collected in patients with severe cardiorespiratory failure treated with ECMO for a period of 3 years (2019–2021). In addition to the presence of pleural fluid, severe lung consolidations with score 4 in the basal lung regions and no better than score 3 in the anterior regions were diagnosed by applying a complex CUS protocol in six regions on both right and left hemithorax [4]. Patients were supine with the trunk elevated at a 15° angle to the horizontal, corresponding to the original method described by the authors in 2006 [1]. The key measurements were taken in expiration with transducer scanning in the transverse plane above the base of the lung in the posterior axillary line at the planned drainage spot (Fig. 1). All drainages were performed by intensivists using the blunt forceps technique, and chest drains were pulled from the trocar into the pleural cavity. Patients with pleural separations of less than 10 mm on the initial scan and/or with an absence of extensive lung consolidations (score 4 to 3) were excluded as well as patients with incomplete aspiration of pleural fluid on post-drainage ultrasound.

After excluding three effusions (5.7%) for incomplete drainage, a total of 50 effusions were evaluated and drained in 42 (27 males and 15 females) patients (age 44 ± 17 years, APACHE II 25.8 ± 6.8, SOFA 11 ± 2.5, height 174 ± 7 cm, body weight 87 ± 20 kg). Twenty-eight patients were on veno-venous ECMO, four on veno-arterio-venous ECMO and ten on veno-arterial ECMO. The calibres of chest drains were 12F (*n* = 33), 16F (*n* = 3), 20F (*n* = 7), 24F (*n* = 1), 28F (*n* = 2), 32F (*n* = 2). The main character of the effusion was exudate (*n* = 25), clear transudate (*n* = 12), sanguinolent (*n* = 8) and haemothorax (*n* = 5). The overall drainage-related incidence of iatrogenic pneumothorax or drainage-related bleeding was zero. All ECMO patients were on a pulmoprotective ventilation (BIPAP, *n* = 30, 60%; PSV, *n* = 20; 40%) with plateau pressures up to 24–26cmH_2_O and PEEP 8–12cmH_2_O.

The pleural fluid volume and pleural separations showed normal distributions according to the Kolmogorov–Smirnov test. The maximum pleural separation (Msep) was 24 ± 7 mm, correlating (all Pearson’s correlation) with separation at the dorsal chest wall (Dsep, 21 ± 9 mm, *r* = 0.88, *p* = 0.0001). The paravertebral (Psep) and lateral (Lsep) separations were 17 ± 8 mm and 17 ± 7 mm, respectively (Fig. 1). The classic [1] method of pleural fluid estimation produced a mean underestimation error of − 359 ± 187 ml, while the mean drained volume was 837 ± 206 ml. The Msep value correlated significantly with drained volume (*r* = 0.47, *p* = 0.001); however, the best correlation was found for the Lsep (*r* = 0.61, *r*^2^ = 0.37, *p* = 0.0001, Fig. 1). The volume of pleural fluid may be estimated with equation V[ml] = 540 + 17*Lsep[mm], resulting in a mean prediction error of 129 ± 98 ml. Similarly, the sum (Ssep) of basal, lateral and ventral pleural separations (mean 55 ± 18 mm) correlated with the drained volume (*r* = 0.54, *p* = 0.0001), showing a mean prediction error of 144 ± 95 ml. Only for the classic method, the prediction bias for the volume estimate was significantly different from zero (Bland–Altman, *p* = 0.0001). Comparison of the right and left pleural effusions did not show a significantly better correlation of Lsep of the right hemithorax (*n* = 30, *r* = 0.66, *p* = 0.0001) compared to the left hemithorax (*n* = 20, *r* = 0.52, *p* = 0.02; *p* = 0.49 for comparison, Fischer´s z-transformation).

The known CUS assessment of pleural fluid volume largely underestimates reality [1] in severely consolidated lungs surrounded by potentially misleadingly thin layer of circumferential effusion. The prediction error may be greatly reduced by calculating with pleural separation above the posterior axillary line which enables better evaluation of the benefits of pleural drainage versus the risks of complications in anticoagulated patients on ECMO (Fig. 1).

Potential sources of error were the variability of mean airway pressures, regardless of a PEEP close to 10cmH_2_O in all patients on ECMO with a protective mode of IPPV and plateau pressures up to 24–26cmH_2_O. The results can also be influenced by interobserver variability which is significantly lower for a pleural separation measurement than for an alveolar-interstitial consolidation assessments [5].

## Data Availability

The data set analysed during the study is available from the corresponding author upon request.

## Abbreviations

ECMO: Extracorporeal membrane oxygenation
CUS: Chest ultrasound
ARDS: Adult respiratory distress syndrome
IPPV: Intermittent positive pressure ventilation
PEEP: Positive end-expiratory pressure
Lsep: Lateral separation of pleural layers
Dsep: Dorsal separation of pleural layers
Psep: Paravertebral separation of pleural layers
Msep: Maximum separation of pleural layers
Ssep: Sum of separation of pleural layers
